# Emotional stimuli candidates for behavioural intervention in the prevention of early childhood caries: a pilot study

**DOI:** 10.1186/s12903-019-0718-4

**Published:** 2019-02-18

**Authors:** Michaela Bartosova, Miroslav Svetlak, Martina Kukletova, Petra Borilova Linhartova, Ladislav Dusek, Lydie Izakovicova Holla

**Affiliations:** 1 Clinic of Stomatology, Institution Shared with St. Anne‘s Faculty Hospital, Faculty of Medicine, Masaryk University, Brno, Czech Republic; 2 Department of Psychology and Psychosomatics, Faculty of Medicine, Masaryk University, Brno, Czech Republic; 3 Department of Pathophysiology, Faculty of Medicine, Masaryk University, Brno, Czech Republic; 4 Institute of Biostatistics and Analyses, Masaryk University, Brno, Czech Republic; 5 RECETOX – Research Center for Toxic Compounds, Faculty of Science, Masaryk University, Brno, Czech Republic

**Keywords:** Behavioural intervention, Dental caries, Early childhood caries, Prevention, Primary dentition

## Abstract

**Background:**

Oral diseases, such as early childhood caries (ECC), have a complex etiology with common, behaviour-related risk factors. Appropriately targeted behavioural intervention using effective tools can help to eliminate risk behaviour leading to ECC. The aim of this study was to ascertain which visual stimuli with a supporting text evoke the strongest emotional response in infants’ mothers and, therefore, are suitable candidates for inclusion in behavioural interventions within the prevention of ECC.

**Methods:**

Thirty-nine mothers of one-year-old children who filled out an originally designed electronic questionnaire, containing 20 visual stimuli with accompanying texts related to dental caries (10/10 with positive/negative intended emotional response), were included in this cross-sectional study. The emotional impact of each stimulus in the mothers was evaluated using the Self-Assessment Manikin (SAM) technique, which represents three emotional dimensions: valence, arousal, and dominance.

**Results:**

Each of the stimuli was assessed by the mothers of infants based on its emotional impact. The real emotional response (evaluated according to the median of valence) was in line with the primarily intended response in 90% of cases (*p* < 0.05). The text with a warning evoked a greater emotional response (evaluated according to the median of arousal) in mothers than only the informative instruction (*p* < 0.05). The relationship between arousal and valence (r = − 0.99; p < 0.05) indicates that the more aversive stimuli raise higher arousal. The significant correlation between valence and dominance shows that the more positive the stimuli, the higher feeling of control over the evoked emotion the mothers have (r = 0.83; *p* < 0.05), and, on the contrary, the lowest control over emotion is correlated with higher arousal (r = − 0.85; p < 0.05). Generally, mothers rated themselves as in high control of their emotions over the individual stimuli.

**Conclusions:**

This pilot study proved that negative pictorial and text warnings about the risks of developing caries had the potential to evoke strong emotional responses in the mothers of infants. We identified three visual stimuli that could be included in future extensive motivation material in an attempt to affect the preventive behaviour of mothers, and thus the oral health of their infants.

**Electronic supplementary material:**

The online version of this article (10.1186/s12903-019-0718-4) contains supplementary material, which is available to authorized users.

## Background

Dental caries is a multifactorial disease caused mainly by the presence of microbial plaque on the teeth surface related to poor oral hygiene, inappropriate dietary habits, and low tooth surface exposure to fluoride [1]. Despite a number of preventive measures, dental caries remains one of the most widespread infectious diseases in the world. Early childhood caries (ECC) is defined as the presence of one or more decayed (non-cavitated or cavitated lesions [d_1_-d_4_]), missing (due to caries), or filled tooth surfaces in any primary tooth in a child under the age of six [2]. Severe ECC (s-ECC) is any sign of smooth-surface caries in a child younger than 3 years of age [2]. Based on the etiopathogenesis of ECC, it is possible to define non-modifiable (genetic and socioeconomic) and modifiable (dietary habits and oral hygiene) factors related to this oral disease. It has been suggested that one of the key factors in the etiology and subsequently the prevention of dental caries, is the behaviour of mothers or other caregivers [3, 4]. Despite satisfactory knowledge of the risk factors for dental caries, information alone is not sufficient to change behaviour and a special behavioural intervention is necessary [5].

Emotions are closely linked to the approach and avoidance motivation strategies which represent the fundamental motivational forces governing human behaviour. They can be viewed as “push” forces that predispose to act in a certain way [6]. Each motivated behaviour can be divided into two tendencies by activating two different brain systems: the tendency to approach a pleasant stimulus (reward system) and the tendency to repulse from an aversive stimulus (defence system) [7]. The general aim of behaviour could be defined as an effort of people to move closer to the desired emotional state. In other words, actions in reaction to emotions should lead to the achievement of a more ‘good for me’ or less ‘bad for me’ state [8]. In order to evoke the person’s motivation to behave in a certain way, the stimulus that is to provoke must be connected with the emotional reaction, in the sense of the basic assessment as to whether it is a good or bad state for the person.

Currently, most information on dental caries prevention is presented as a professional recommendation in a rather emotionally neutral textual form. The “evidence-based” recommendation produces positive emotions in health care providers, for others it is rather neutral and incomprehensible. Although verbal expressions of emotions and feelings in humans are very variable, factor-analytic studies of emotional language have concluded that each stimulus can be placed into a three-dimensional affective space which is defined by: emotional valence, emotional arousal and dominance [9]. These dimensions are closely connected with the motivation of behaviour. The coordinates of the stimulus (e.g., photograph, text, sound, etc.) in the affective space, expressed by valence, arousal and dominance, represent important information about its motivational power.

In this context, searching for effective approaches to motivate people to change risk behaviour or to create health-promoting behaviour, is a major challenge of preventive medicine.

## Methods

The aim of this pilot study was to ascertain which visual stimuli with a supporting text evoke the strongest emotional response in infants’ mothers and, therefore, are suitable candidates for inclusion in behavioural interventions within the prevention of ECC.

The originally designed electronic questionnaire (Additional files 1 and 2) contained 20 stimuli, which represented the risk factors of dental caries in children or showed the appropriate behaviour of mothers in the care of their children’s dentition. The visual stimuli (10 with an intended positive response and 10 with an intended negative response), accompanied by verbal descriptions, warnings or information (instruction), were designed in line with the recommendations of the European Academy of Pediatric Dentistry [1]. The visual stimuli were chosen according to the experience of the clinicians from our out-patients clinic. The most serious conditions were included in aversive pictures. The positive picture set was composed of the most desired aims of prevention of dental caries (pictures 2, 4, 6, 8, 10, 12, 14, 16, 18, and 20).

**Fig. 1:**
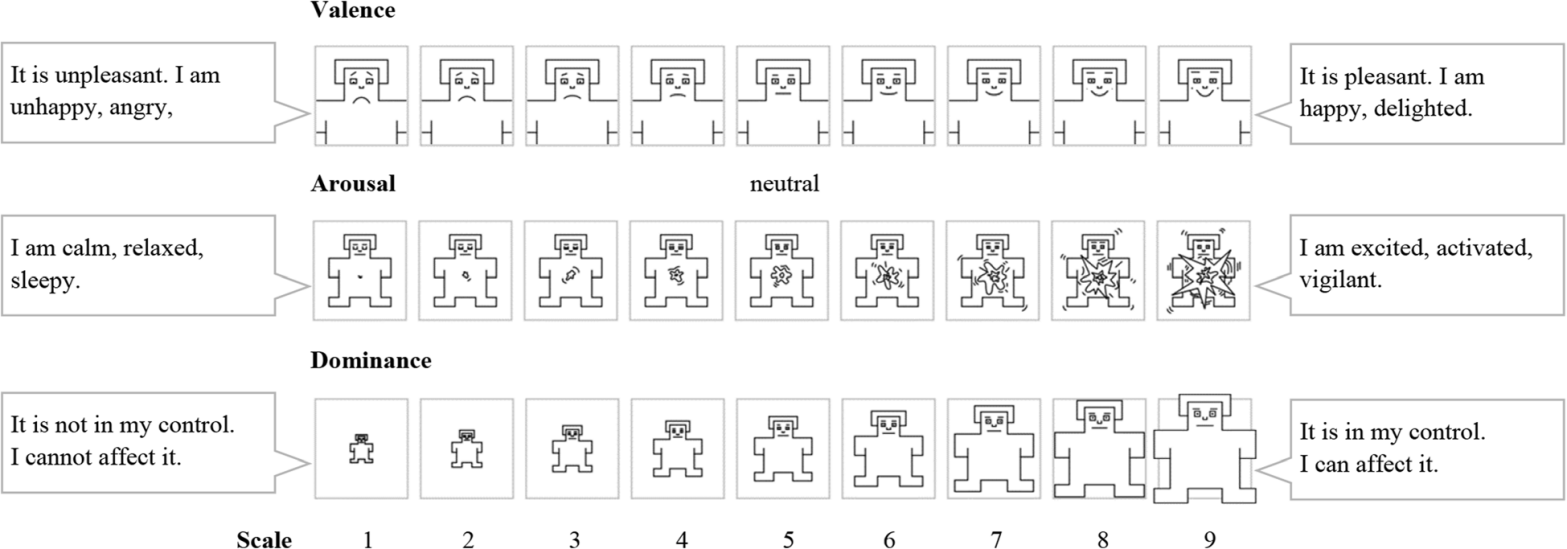
Self-Assessment Manikin scale [18]

The emotional impact of these 20 individual stimuli on mothers was evaluated using the Self-Assessment Manikin (SAM) method (Fig. 1) [10]. The SAM graphical scale is a tool for the assessment of the three basic emotional dimensions: valence, arousal, and dominance. The emotional valence (first line in Fig. 1) is a bipolar scale which describes a continuum between polarities that are extremely unpleasant (unhappy manikin on the left) and extremely pleasant (happy manikin on the right). The scale ranges from 1 to 9 (1 extremely unpleasant, 5 neutral, 9 extremely pleasant). The arousal scale (the second line in Fig. 1) represents a continuum which ranges from 1 (calm, relaxed manikin on the left) to 9 (extremely excited, aroused manikin on the right). The dominance scale (third line in Fig. 1) describes the level of subjectively referred sense of control over the emotion evoked by the stimulus. It ranges from 1 (loss of control, manikin on the left) to 9 (full control, manikin on the right). The subject is instructed to evaluate their immediate subjective feeling being evoked by the test material (texts and pictures in our study) across the three afore-mentioned dimensions.

During the period from 04/2017 to 10/2017, 97 mothers of one-year-old children were addressed with a request to join this cross-sectional study. Ninety-seven infants were examined with a dental mirror and probe by one pediatric dentist at the Clinic of Stomatology, Faculty of Medicine, Masaryk University and St. Anne’s Faculty Hospital. The d_1_mft index was calculated using dental caries (d_1_ level) as a cut-off point for the detection of decay. There were no exclusion criteria for mothers and infants. Only 39 mothers signed informed consent forms and agreed to participate in our study. The basic demographic characteristics (age of mothers, education of both parents), and their awareness of caries risk factors and dental caries prevention, i.e. suitable diet, drinking sweet beverages, significance of teeth cleaning/brushing, suitable teeth brushing techniques, observance of oral health of their children etc., were determined in all of them. Subsequently, after being instructed as to how to record the emotional perception evoked in them by a stimulus on the SAM scale, (see Additional file 1), the mothers were asked to fill out the electronic questionnaire. The link to the electronic questionnaire was sent to mothers’ e-mail addresses, the questionnaire was filled out online.

For statistical purposes, the scope of the SAM technique was converted to numbers ranging from 1 to 9. The software Statistica v. 13 (IBM Corporation, 2013) was used for the data analysis. The relationships between the 3 dimensions (valence, arousal, dominance) were computed using Spearman’s correlation coefficient (r). For the description of variables the mean and standard deviation (SD), median and interquartile range (IRQ), and minimum and maximum were used. Statistical significance of relationship between categorical variables was tested using Fisher exact test, statistical significance of differences in ordinal scores between groups was tested using Mann Whitney U test. The sample size was planned on the basis of expected width of 95% confidence interval for average score estimate with expected standard deviation of pseudo-continuous scoring scale 1. The acceptable width for the 95% confidence interval of average score estimate was set as 0.7; it corresponds to *N* = 35. The computation of the overall sample size was estimated using previously experienced response rate approximately 30–40% in similar studies [11–13]. The response rate to the questionnaires was estimated to be assumed to be approximately 40%, thus 97 mothers were addressed to reach 35–40 responses.

## Results

In this pilot study, 39 mother-child pairs were included. The electronic questionnaire response rate was 40.2%. 35.9% of mothers were in the age group of 21–30 years and 64.1% were in the age group of 31–40 years. 51.3% mothers had higher (university) education. Only four mothers had lower than secondary school education. The mean age ± standard deviation (SD) of the infants was 11.2 ± 2.4 months (median age [interquartile range, minimum-maximum] 10.0 [10.0–12.0, 8.0–20.0] months) with 5.6 ± 3.8 (5.0 [3.0–7.0, 0–16.0]) primary teeth. The examination revealed beginning of caries in three children (d_1_mft) on all upper incisors, i.e. d_1_mft = 4, the other children were caries free.

Five mothers reported that they did not use tooth paste for cleaning their infants’ teeth, only two mothers served sweetened beverages to their children and none of them dipped a pacifier into something sweet before giving it to their child. Up to 28.2% of mothers cleaned a pacifier in their mouths and even 43.6% of them occasionally or regularly tasted a meal with the same spoon or licked the spoon while feeding the infant.

Each of the stimuli in the electronic questionnaire was defined based on its emotional impact on the infants’ mothers. Table 1 shows the statistical evaluations (median and interquartile range) of valence, arousal, and dominance produced by ranking the individual stimuli in the questionnaire. There was a highly statistically significant relationship between the expected and observed emotional result of the visual stimuli (10 negative and 10 positive vs. 8 negative and 12 positive, *p* < 0.05). The real emotional response (evaluated according to the median of valence) was in line with the primarily intended response in 90% of cases. Highly statistically significant difference in the median values of valence between groups defined according to the expected negative and positive emotional result was observed (10 intended negative emotional stimuli with median of valence 2 [IQR, 2–4] and 10 intended positive emotional stimuli with valence 9 [IQR, 8–9], *p* < 0.05). The text with a warning evoked a greater emotional response in mothers than only the informative instruction. The significant difference in median values of arousal between groups categorized according to the type of text was found (8 text warnings with median of arousal 6 [IQR, 5–7] and 12 text instructions with arousal 2 [IQR, 1–2], *p* < 0.05).

**Table 1:** Ranking of the stimuli in the questionnaire (valence, arousal, dominance)

S	V	T	Text	Median (IQR)			
				Valence	Arousal	Dominance	R
*5*	N	W	Lack of mother’s care for her infant’s teeth causes tooth decay.	*1 (1–2)*	*8 (5–9)*	8 (7–9)	N
*13*	N	W	Neglecting care of your child’s teeth leads to serious complications.	*1 (1–3)*	*7 (5–9)*	8 (6–9)	N
*7*	N	W	Untreated tooth can also endanger your child’s life.	*2 (1–3)*	*7 (5–9)*	8 (6–9)	N
15	N	W	Caries hurt children.	*2 (1–3)*	7 (3–8)	7 (5–9)	N
17	N	W	Poor dentition requires anesthesia.	*2 (1–5)*	6 (3.5–8)	8 (5–9)	N
9	N	W	By neglecting regular care at the dentist, you expose your child to an unpleasant treatment.	*3 (2–5)*	6 (3–8)	7 (7–9)	N
3	N	W	Sleeping with a bottle increases the risk of tooth decay.	3 (2–7)	4 (2–7)	9 (7–9)	N
19	N	W	Mother may be a source of bacteria supporting tooth decay in a child.	4 (2–7)	5 (3–7)	7 (5–9)	N
20	P	I	By avoiding kissing baby on the lips, you reduce the risk of transmission of bacteria that cause tooth decay.	6 (3–9)	3 (1–6)	8 (6–9)	P
1	N	I	We do not offer sweetened drinks to children.	7 (4–9)	3 (2–5)	9 (7–9)	P
16	P	I	Preventive examination takes place twice a year.	8 (5–9)	2 (1–5)	9 (8–9)	P
4	P	I	A visit to a dentist can be painless.	8 (5.5–9)	2 (1–5)	7 (6.5–9)	P
11	N	I	Regular dental examinations allow the dentist to detect dental caries in time and treatment is painless.	8 (6–9)	2 (1–4)	9 (8–9)	P
14	P	I	We clean our child’s teeth twice a day.	8 (7–9)	2 (1–3)	9 (7–9)	P
2	P	I	Parents must assist their child with care for their teeth till the child is six.	9 (7–9)	2 (1–4)	9 (7–9)	P
6	P	I	At the age of 1 year, child eats from his/her own saucer and with his/her own cutlery.	9 (7.5–9)	2 (1–4)	8 (6.5–9)	P
18	P	I	Regular care protects your child’s teeth.	9 (8–9)	1 (1–3)	9 (8–9)	P
8	P	I	Drinking pure water reduces the risk of tooth decay in children.	9 (8–9)	1 (1–2)	9 (8–9)	P
12	P	I	Parents are the ultimate model for children.	9 (8–9)	1 (1–2)	9 (8–9)	P
10	P	I	Regular care protects your child’s teeth.	9 (8–9)	1 (1–2)	9 (8–9)	P

The relevant statements are ordered according to their valence, from the most negative to the most positive. *The combination of visual and text stimuli with the highest potential for the use in behavioural interventions.* Numbers in italics represent stimuli assessed by more than 75% of mothers with valence ≤5/ arousal ≥5. S = stimulus in the questionnaire, V = intended visual stimulus, T = text stimulus, R = emotional response of mothers to visual and text stimuli, IQR = interquartile range, N = negative, P = positive, W = warning, I = information

**Fig. 2:**
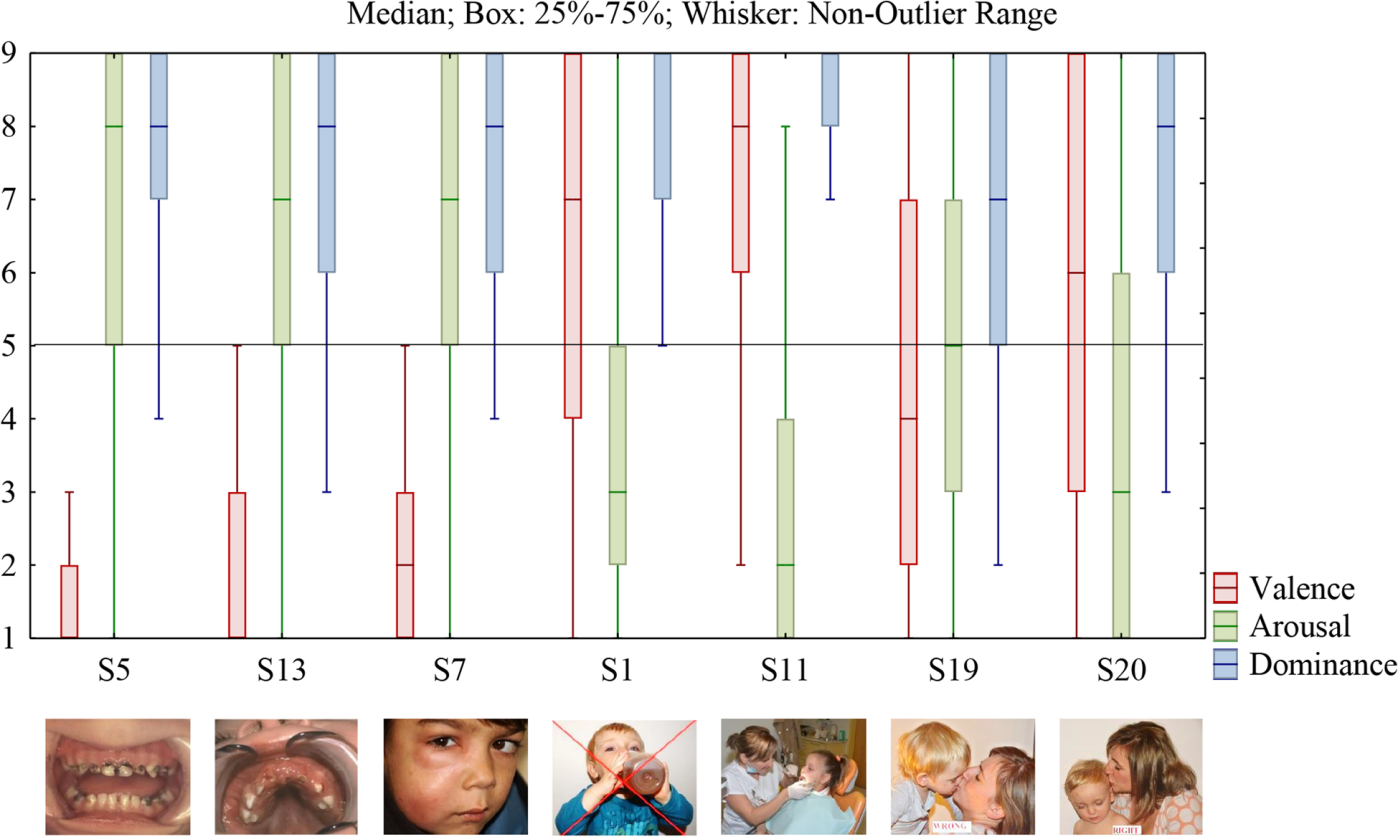
Ranking of selected stimuli

Stimulus 5 was perceived as the most negative with the lowest valence of 1 and the highest arousal of 8, with 95% of the mothers giving the picture and the description a negative valence (valence less than 5) and 69.2% high arousal. Stimuli 3, 5, 7, 9, 13, 15, 17, and 19 exhibited low valence (median from all texts and pictures ≤5) and can be considered as negative. High arousal (median of all statements ≥5) was recorded only in stimuli 5, 7, 9, 13, 15, 17, and 19. Negative stimuli 5, 7, and 13 including text warnings were assessed by more than 75% of mothers with a valence ≤5 and at the same time with an arousal ≥5 (Fig. 2).

Our results demonstrated that not all stimuli originally intended as negative were perceived by the infants’ mothers adversely. Negative stimuli 1 and 11 were evaluated by mothers with valence 7 and 8 (respectively), and thus as positive. However, stimuli 19 and 20 with an intended opposite response were evaluated by mothers as we had presumed, i.e. they evoked a rather neutral valence and a mean value of arousal (Fig. 2).

It is evident that the exposure of mothers to stimuli did not cause a significant loss of control over the evoked emotions (see Table 1). The median value of all responses was higher than 5 in all cases. For this reason, only the results relating to the remaining two subscales (i.e. valence and arousal) are further presented.

**Fig. 3:**
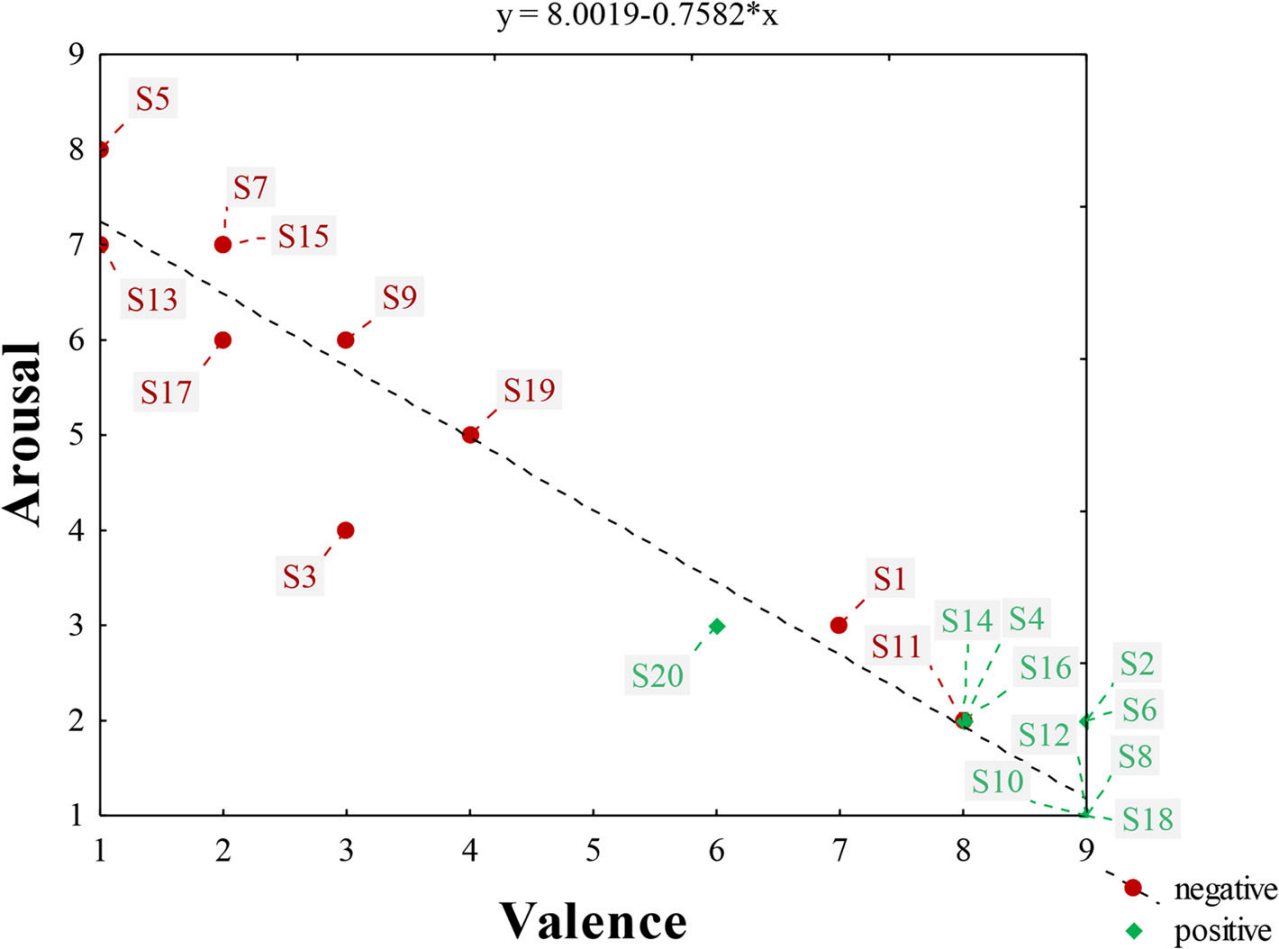
Two-dimensional chart of mother’s emotional response to 10 positive and 10 negative stimuli

All positive images and descriptions generated positive valence and low arousal (Fig. 3). Stimuli 8, 10, 12, and 18 (with median of valence 9 and arousal 1) were evaluated as the most positive ones. The relationship between arousal and valence (*r* = − 0.99; *p* < 0.05) indicates that the more aversive stimuli raise higher arousal. The significant correlation between valence and dominance shows that the more positive the stimuli, the higher feeling of control over the evoked emotion the mothers have (*r* = 0.83; *p* < 0.05), and, on the contrary, the lowest control over emotion is correlated with higher arousal (*r* = − 0.85; *p* < 0.05). Generally, mothers rated themselves as in high control of their emotions over the individual stimuli.

## Discussion

Several studies have focused on behavioural interventions within prevention of ECC and other oral diseases. Behavioural interventions to reduce caries have been based on a variety of behaviour change theories and approaches − mainly the social cognitive theory and the related health belief model and the theory of planned behaviour, self-determination theory, and motivational interviewing [14, 15].

The preventive programs are primarily aimed at children at primary schools [16]. Programs trying to influence parental behaviour are rare [17]; they mostly affect the frequency of tooth cleaning but do not lead to a change of dietary habits in children [17]. We believe that intervention within prevention of ECC should be targeted at mothers as they play an important role in health behaviour of the whole family, especially infants.

As already mentioned, emotions are an important part of the motivation to change behaviour. Triggering positive or negative emotions associated with problematic behaviour is one of the fundamental processes of this change [18]. The most effective approaches in preventive medicine are those with minimal economic costs and maximum population impact [19]. Searching for stimuli that evoke strong emotions and address the relevant target groups is a key step of each effective preventive intervention.

This also was the aim of the submitted pilot study as the stimulus material for the intervention approach for the Czech population has not been formed and standardized yet. Stimuli 5, 7, and 13 in our questionnaire have the lowest valence and the highest potential to evoke a strong emotional response in the mothers of infants and thus may be used as aversive stimuli within behavioural interventions in the field of oral health. The highest arousal was evoked by a picture of the damaged teeth of a child and the statement “Lack of mother’s care for her child’s teeth causes tooth decay.” The high dominance associated with all stimuli (including stimulus 5) could be explained again by the fact that the experimental group probably consisted of responsible mothers, who visit the dentist regularly and have no reason to worry.

While the negative pictures with warnings were evaluated by mothers as we had presumed, both negative figures with educational texts were evaluated with an unexpectedly high valence. In stimulus 1, this can also be explained by the fact that the crossed-out positive figure does not evoke negative emotions. In stimulus 11, the figure shows a child being treated by a dentist, similarly as in stimuli 4, 9, 16, and 17. We suppose that the mothers of infants in our studied group perceived the dental treatment neutrally and thus these types of figures should be replaced by other images in following studies.

Nevertheless, stimuli 1 and 11 represent an interesting subgroup of pictures with supporting texts in terms of the emotional responses of mothers. The responses to them very likely depend on the behaviour of the mothers and thus constitute a continuum between positive and negative valence reactions. Mothers participating in this study cared for their children’s oral health and generally maintained preventive measures. Therefore, they appreciated this information as positive and might be willing to follow the recommendations. Negative responses could be expected from the mothers on the opposite side of the continuum whose behaviour is unhealthy and negative pictures with texts confront them with this fact.

In general, current prevention medicine uses only a limited repertoire of behaviour change techniques [20, 21]. It mostly focuses on providing information on the relationship between behaviour and health or fear appeal. For example, the global prevention campaign on tobacco products is based on aversive visual stimuli and text warnings. However, it has been repeatedly shown that education and intimidation are not enough [14] and that people are more motivated by moving towards positive emotions [22] than by avoiding negative situations. The approach often neglected by a contemporary biomedical model of medicine is based on the premise that rather than avoiding fear it is more natural for people to move towards some appreciated personal values and principles (e.g.,“to be a good mother”). A preventive campaign based on this principle should be the next focus of research into the prevention of tooth decay.

For this reason, positive stimuli were also included in our questionnaire, for example a picture of a mother who is cleaning her teeth with her child and the sentence: “Parents are the ultimate model for children”. This sentence was rated by mothers as one of the most positive, but with very low arousal. Finding that positive images are associated with lower arousal agrees with the results of other studies [23]. It is still unclear what role arousal plays in the positive motivation and what its optimal level for the most effective behavioural change should be.

The warning or information about the inappropriateness of kissing a child on the mouth (stimuli 19 or 20) due to the possible transfer of the cariogenic bacteria from the mother’s mouth into the child’s oral cavity evoked a rather neutral valence and a mean value of arousal, whereas the interquartile range is greatest at the valence of these stimuli. It probably reflects great ambivalence of mothers to this statement. The picture of mother kissing her child with information about a possible transmission of bacteria has the potential to be used as a positive stimulus in the intervention process.

The main limitation of this study is a low number of probands due to a lack of interest across a group of mothers of infants. The fact that the electronic questionnaire was filled out by the mothers online may have played a role in the low response rate. Mothers were not under pressure to participate. On the other hand, responders should be in emotional stable state when filling out the electronic questionnaire. The final number of the obtained questionnaires generated a sample size which was sufficient for the planned statistical estimates with a relevant confidence interval.

Another problem is the significant impact of selection, especially when mothers with higher motivation and generally better knowledge of risk factors of tooth decay (“conscious mothers”) were willing to be involved in the study. There is a high probability that mothers with risk behaviour were not interested in participating in the questionnaire survey. Further research will need to be done on a larger sample of the population, including mothers from risk groups.

Our constant hypothesis is that the stimulus material should firstly impress mothers with a negative warning, thus developing negative emotions in the form of concerns about their child’s health, and at the same time offer them a solution as to how to avoid the feared consequences of their behaviour. In the future follow-up study, we intend to create a portfolio of emotionally-charged stimuli (images with texts) and then verify their effectiveness in behavioural intervention. The stimuli material can be a beta version for a motivational interview or a smart phone application which we will test on the Czech population. We can draw inspiration from the studies in the population of the American Indians [24] or studies by Nolen et al. [25]. It will be necessary to determine the percentage of mothers who really have changed their behaviour on the basis of the given prevention method (reduced consumption of sweetened drinks, regular preventive examinations, etc.) and to verify whether the defensive mechanisms such as generalization or rationalization were not activated.

## Conclusions

This pilot study proved that negative pictorial and text warnings about risks of developing caries had the potential to evoke strong emotional responses in the mothers of infants. We identified three visual stimuli which could be included in a future extensive motivation material in an attempt to affect the preventive behaviour of mothers, and thus the oral health of their infants. Future research in this area should focus on creating extensive stimulus material and its subsequent verification on a large population.

## Additional files

Additional file 1: Instructions for filling out the electronic questionnaire (PDF 124 kb)

Additional file 2: Overview of the stimuli in the electronic questionnaire (S1-S20) (PDF 364 kb)

## Abbreviations

d: decay, part of d_1_mft decay/missing/filled teeth index
ECC: early childhood caries
I: information
IQR: interquartile range
N: negative
P: positive
p: *p*-value
r: correlation coefficient
R: emotional response of mothers to visual and text stimulus
S: stimulus in the questionnaire
SAM: Self-Assessment Manikin
SD: standard deviation
s-ECC: severe form of early childhood caries
T: text stimulus
V: intended visual stimulus
W: warning
