# Supplementary material for: Emotional stimuli candidates for behavioural intervention in the prevention of early childhood caries: a pilot study
Source: BMC Oral Health. 2019 Feb 18;19:33. doi: 10.1186/s12903-019-0718-4 (PMC6379970; doi:10.1186/s12903-019-0718-4)

Instructions for filling out the electronic questionnaire

In the following questionnaire, you will find a set of twenty pictures with accompanying text that relates to your child's oral cavity health. Your task is to look at each picture, read the accompanying text and then evaluate the feeling that the picture and text evoke in you. To do this, select and mark with a cross the manikin in each of the three lines that best suits your current feeling. The scale is explained in the picture below.

Thank you for your collaboration.

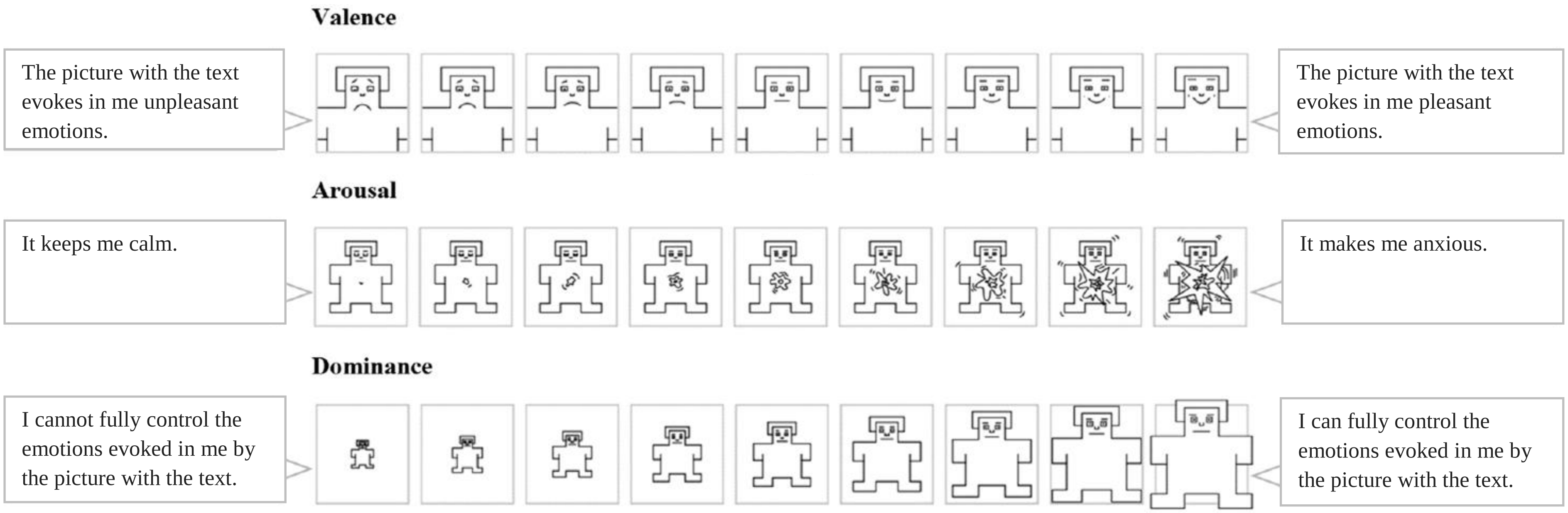

Supplement: Supplementary file 1 — Instructions for filling out the electronic questionnaire (PDF 124 kb) [file 12903_2019_718_MOESM1_ESM.pdf]
