# Supplementary material for: Emotional stimuli candidates for behavioural intervention in the prevention of early childhood caries: a pilot study
Source: BMC Oral Health. 2019 Feb 18;19:33. doi: 10.1186/s12903-019-0718-4 (PMC6379970; doi:10.1186/s12903-019-0718-4)
Supplement: Supplementary file 2 — Overview of the stimuli in the electronic questionnaire (S1-S20) (PDF 364 kb) [file 12903_2019_718_MOESM2_ESM.pdf]

**S1**	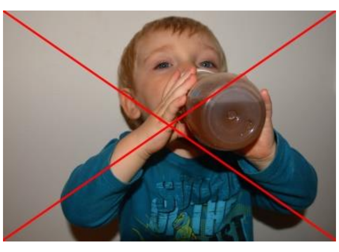	**We do not offer sweetened drinks to the child.**
**S2**	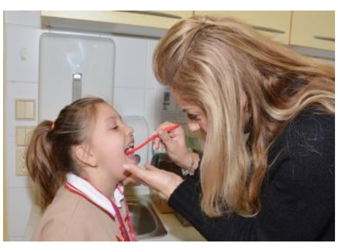	**Parents must assist their children with care for their teeth till the children are six.**
**S3**	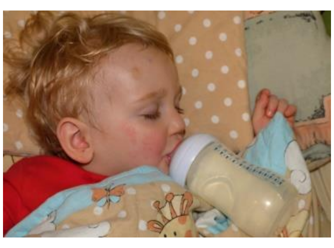	**Sleeping with a bottle increases the risk of tooth decay.**
**S4**	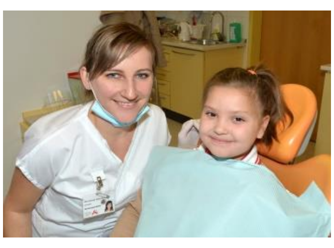	**A visit to a dentist can be painless.**
**S5**	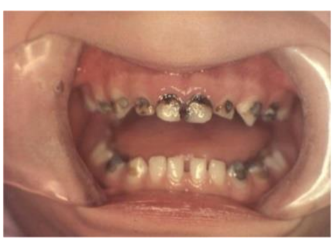	**Lack of mother’s care for her infant's teeth will cause tooth decay.**
**S6**	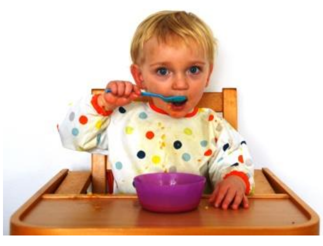	**At the age of one year, child eats from his/her own saucer and with his/her own cutlery.**
**S7**	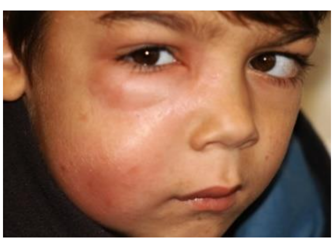	**Untreated tooth can also endanger your child's life.**
**S8**	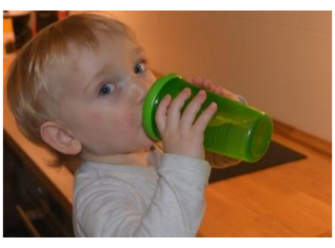	**Drinking pure water reduces the risk of tooth decay in children.**
**S9**	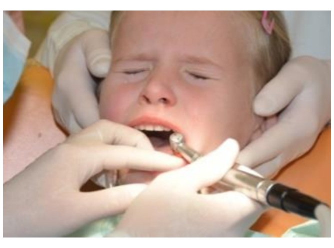	**By neglecting regular care at the dentist, you expose your child to an unpleasant treatment.**
**S10**	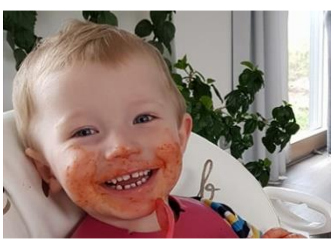	**Regular care protects your child's teeth.**
**S11**	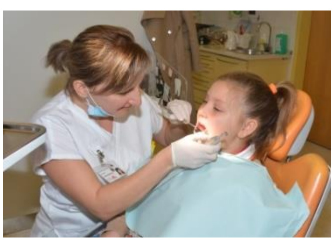	**Regular dental examinations allow the dentist to detect dental caries in time and treatment is painless.**
**S12**	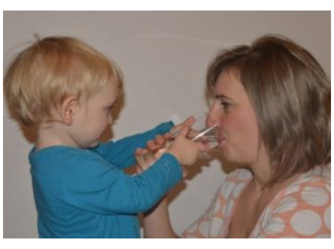	**Parents are the ultimate model for children.**
**S13**	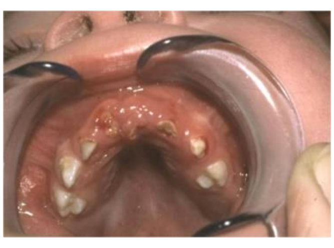	**Neglecting care of your child's teeth leads to serious complications.**
**S14**	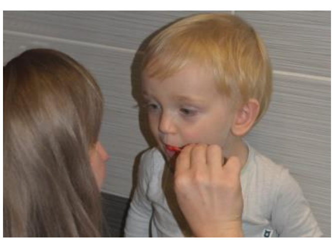	**We clean our child's teeth twice a day.**
**S15**	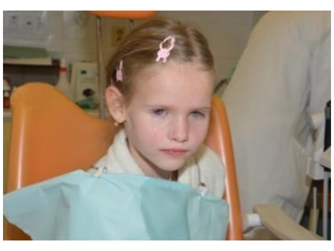	**Caries hurt children.**
**S16**	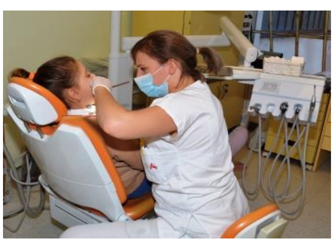	**Preventive examination will take place twice a year.**
**S17**	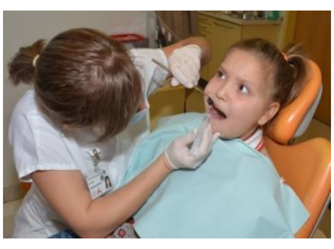	**Poor dentition requires anesthesia.**
**S18**	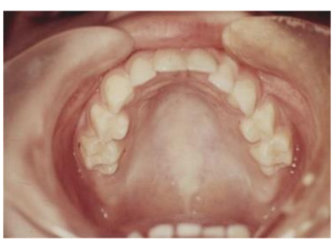	**Regular care protects your child’s teeth.**
**S19**	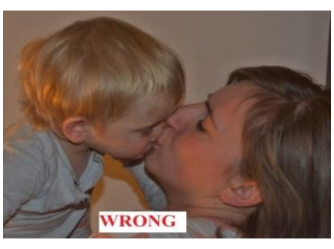	**Mother may be a source of bacteria supporting tooth decay in a child.**
**S20**	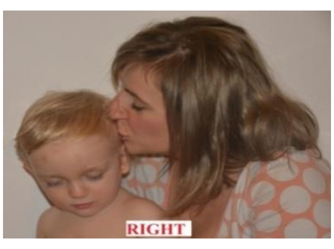	**By avoiding kissing baby on the mouth, you reduce the risk of transmission of bacteria that cause tooth decay.**
